# Complete mitochondrial genome and phylogenetic analysis of *Callorhinus ursinus*: an endangered species from South Korea

**DOI:** 10.1080/23802359.2018.1457992

**Published:** 2018-04-23

**Authors:** Seon-Mi Lee, Mu-Yeong Lee, Hey Sook Jeon, Jung A. Kim, Sang-Hwa Lee, Junghwa An

**Affiliations:** a BK21 PLUS Program for Creative Veterinary Science Research and Conservation Genome Resource Bank for Korean Wildlife (CGRB), College of Veterinary Medicine, Seoul National University, Seoul, Republic of Korea;; b DNA Analysis Division, Seoul Institute, National Forensic Service, Seoul, Republic of Korea;; c Animal Resources Division, National Institute of Biological Resources, Incheon, Republic of Korea;; d Graduate Program in Cellular Biology and Genetics, College of Medicine, Chungbuk National University, Cheongju, Republic of Korea

**Keywords:** *Callorhinus ursinus*, mitochondrial genome, northern fur seal, next generation sequencing

## Abstract

We present the complete mitochondrial genome and a phylogenetic analysis of *Callorhinus ursinus*, the northern fur seal, determined using Illumina next-generation sequencing (NGS) technology. The total length of the mitogenome was 17,154 bp, which consisted of 13 protein-coding genes, two ribosomal RNA genes, 22 tRNA genes, and one control region. The base composition of the entire mitogenome was 33.5% (A), 26.3% (C), 13.9% (G), and 26.3% (T) with an A + T bias of 59.8%. The control region contained two types of tandem repeats. A neighbour-joining (NJ) tree was constructed and comprised two clades with *C. ursinus* forming a monophyletic group. Data produced in this study will aid exploration of the genetic diversity of endangered *C. ursinus* and contribute to molecular identification of this species.

The northern fur seal, *Callorhinus ursinus*, belongs to the family Otariidae and the order Carnivora (King 1991). This species is pelagic and is found in the North Pacific Ocean from the Sea of Okhotsk to the northern Bering Sea and as far south as 34°N (Gentry 1998). In the past, the northern fur seal declined due to entanglement in fishing nets and hunting for the fur trade (Trites 1992). Therefore, it has been internationally classified as vulnerable on the Red List of Threatened Species by the International Union for the Conservation of Nature and as an endangered species II by the Ministry of Environment in Korea.

The complete mitochondrial genome of an organism is a valuable source of information and can contribute to inferring the phylogenetic relationships of various taxa more accurately and with more detail than can be done with short sequences (Douglas and Gower 2010; Peng et al. 2016; Yu et al. 2016). Here, we determined the mitogenome sequence of *C. ursinus* from South Korea and compared it with a previously reported one for this species and those of other species in the family Otariidae.

Muscle tissue (IN1671) of *C. ursinus* was collected from individuals in Samcheok-si, Gangwon-do, South Korea. The specimen and DNA were deposited at National Institute of Biological Resources at Incheon, South Korea. Total genomic DNA was isolated using a DNeasy^®^ Blood & Tissue Kit (Qiagen, Valencia, CA) following the manufacturer’s instructions, and next-generation sequencing (NGS) was performed with an Illumina HiSeq 2500 platform at the National Instrumentation Center for Environmental Management, Seoul, South Korea. Annotation of protein-coding genes (PCGs), ribosomal RNAs (rRNAs), and transfer RNA (tRNA) genes was conducted using the online tool DOGMA (Wyman et al. 2004) and the software ARWEN (Laslett and Canbäck 2008). The complete mitogenome (17,154 bp) of *C. ursinus*, which consisted of 13 PCGs, two rRNAs, 22 tRNA genes, and one control region (D-loop) as in other typical vertebrate mitogenomes (Sorenson et al. 1999), was deposited into GenBank (accession number MG916809). The overall base composition was 33.5% (A), 26.3% (C), 13.9% (G), and 26.3% (T) with an A + T bias of 59.8%. The putative D-loop (1695 bp), located between tRNA-*Phe* and tRNA-*Pro*, contained two types of tandem repeats.

Phylogenetic analysis of the *C. ursinus* mitogenome was performed by comparing it with 13 PCG sequences derived from the mitogenomes of the other six species in the family Otariidae and one from the same species. The neighbour-joining (NJ) method was used to construct a tree with the software MEGA 6.0 (Kumar et al. 2016) using the Kimura 2-parameter model with 1000 bootstrap replicates. The NJ tree had two clades: *C. ursinus* formed a monophyletic group, and the remaining six species belonged to the other clade (Figure 1). Data produced in this study will aid exploration of the genetic diversity of endangered *C. ursinus* and contribute to the molecular identification of this species.

**Figure 1.**
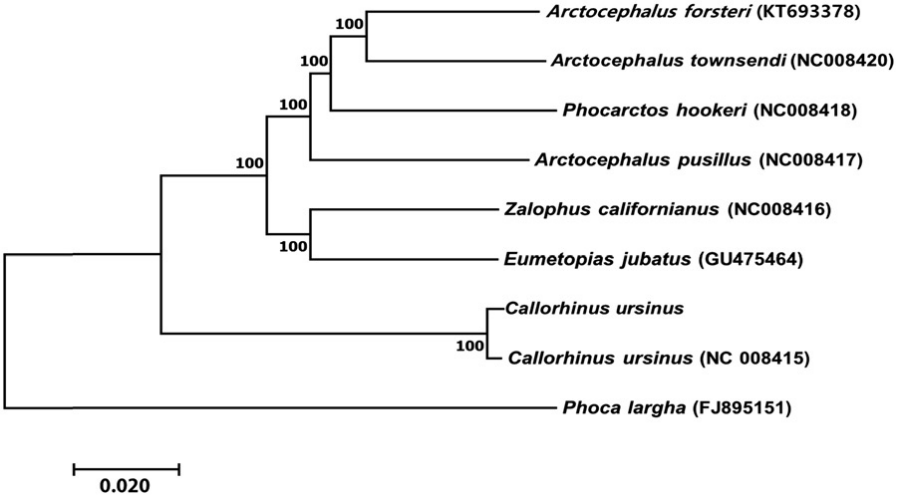
Neighbour-joining tree of seven species of Otariidae based on the concatenated nucleotide sequences of the 13 protein-coding genes from the mitogenomes of each species. Bootstrap values are shown at the nodes. GenBank accession numbers for the sequences are indicated next to species designations.

## References

[CIT0001] Douglas DA, Gower DJ. 2010 Snake mitochondrial genomes: phylogenetic relationships and implications of extended taxon sampling for interpretations of mitogenomic evolution. BMC Genomics. 11:14–14. 20055998 10.1186/1471-2164-11-14 PMC2820454

[CIT0002] Gentry RL. 1998 Behavior and ecology of the Northern Fur Seal. Princeton: Princeton University Press.

[CIT0003] King JE. 1991 Seals of the world. Ithaca: Cornell University Press.

[CIT0004] Kumar S, Stecher G, Tamura K. 2016 MEGA7: molecular evolutionary genetics analysis Version 7.0 for bigger datasets. Mol Biol Evol. 33:1870–1874. 27004904 10.1093/molbev/msw054 PMC8210823

[CIT0005] Laslett D, Canbäck B. 2008 ARWEN: a program to detect tRNA genes in metazoan mitochondrial nucleotide sequences. Bioinformatics. 24:172–175. 18033792 10.1093/bioinformatics/btm573

[CIT0006] Peng L-F, Yang D-C, Lu C-H. 2016 Complete mitochondrial genome of oriental magpie-robin *Copsychus saularis* (Aves: Muscicapidae). Mitochondrial DNA B. 1:21–22. 10.1080/23802359.2015.1137802 PMC7800930 33473393

[CIT0007] Sorenson MD, Ast JC, Dimcheff DE, Yuri T, Mindell DP. 1999 Primers for a PCR-based approach to mitochondrial genome sequencing in birds and other vertebrates. Mol Phylogenet Evol. 12:105–114. 10381314 10.1006/mpev.1998.0602

[CIT0008] Trites AW. 1992 Northern fur seals: why have they declined? Aquat Mamm. 18:3–18.

[CIT0009] Wyman SK, Jansen RK, Boore JL. 2004 Automatic annotation of organellar genomes with DOGMA. Bioinformatics. 20:3252–3255. 15180927 10.1093/bioinformatics/bth352

[CIT0010] Yu P, Ding S, Yang Q, Bi Z, Chen L, Liu X, Song X, Wan Q. 2016 Complete sequence and characterization of the paradise fish *Macropodus erythropterus* (Perciformes: Macropodusinae) mitochondrial genome. Mitochondrial DNA B. 1:54–55. 10.1080/23802359.2015.1137820 PMC7800590 33473406

